# Trends in prenatal diagnosis of congenital anomalies in Western Australia between 1980 and 2020: A population‐based study

**DOI:** 10.1111/ppe.12983

**Published:** 2023-05-04

**Authors:** Cassandra MacArthur, Michele Hansen, Gareth Baynam, Carol Bower, Erin Kelty

**Affiliations:** ^1^ School of Population and Global Health The University of Western Australia Crawley Western Australia Australia; ^2^ Telethon Kids Institute The University of Western Australia Crawley Western Australia Australia; ^3^ Western Australian Register of Developmental Anomalies King Edward Memorial Hospital Perth Western Australia Australia; ^4^ Faculty of Health and Medicine, Institute and Division of Paediatrics University of Western Australia Crawley Western Australia Australia; ^5^ Rare Care, Clinical Centre of Expertise for Rare and Undiagnosed Diseases Perth Children's Hospital Perth Western Australia Australia

**Keywords:** congenital abnormalities, pregnancy, prenatal diagnosis

## Abstract

**Background:**

Advances in screening and diagnostics have changed the way in which we identify and diagnose congenital anomalies.

**Objective:**

To examine changes in rates of prenatal diagnosis of congenital anomalies over time and by demographic characteristics.

**Methods:**

We undertook a population‐based retrospective cohort study of all children born in Western Australia between 1980 and 2020 and diagnosed with a congenital anomaly. Age at diagnosis (prenatal, neonatal, infancy, early childhood or childhood) prevalence (all‐type and type‐specific), and prevalence ratios (PR) were calculated. We fit joinpoint regression models to describe the average annual percentage change (APC) in prenatal diagnosis over time, and log‐binomial regression models to estimate the association between prenatal diagnosis and demographic characteristics.

**Results:**

Prenatal diagnosis prevalence between the first (1980–1989: 28.3 per 10,000 births) and last (2005–2014: 156.1 per 10,000 births) decades of the study increased 5.5‐fold (95% confidence interval [CI] 5.0, 5.9). Substantial increases were observed for cardiovascular (PR 10.7, 95% CI 8.0, 14.6), urogenital (PR 10.5, 95% CI: 8.7, 12.6) and chromosomal anomalies (PR 7.0, 95% CI 5.9, 8.3). Prenatal diagnosis was positively associated with the birth year (adjusted risk ratio [RR] 1.04, 95% CI 1.03, 1.04), advanced maternal age (RR 1.14, 95% CI 1.11, 1.18), multiple anomalies (RR 2.86, 95% CI 2.77, 2.96) and major anomalies (RR 3.75, 95% CI 3.36, 4.19), and inversely associated with remoteness (RR 0.89, 95% CI: 0.83, 0.95) and Aboriginality (RR 0.90, 95% CI 0.83, 0.97).

**Conclusions:**

Increases in prenatal diagnosis of congenital anomalies were observed in Western Australia from 1980 to 2020, reflecting advances in screening. Prenatal diagnosis was less common in remote regions and in Aboriginal children, strengthening calls for increased provision of antenatal care services for these populations.

**Synopsis**

**Study Question**

Has the prenatal diagnosis of congenital anomalies changed since 1980?

**What's Already Known?**

Prenatal diagnosis prevalence is an accepted public health indicator to measure the effectiveness of prenatal screening programs in detecting cases of congenital anomaly. For surveillance registries with case ascertainment periods beyond the neonatal period, age at diagnosis is a useful estimate for identifying late‐diagnosed anomalies that may benefit from screening evaluation.

**What this Study Adds**

Considerable gains have been made the prenatal diagnosis of congenital anomalies overall and by major anomaly types. Prenatal diagnosis prevalence estimates for the Western Australian population are considerably higher than those reported from Europe, though disparities in prenatal diagnosis from differences in remote residence and Aboriginality persist.

## 1 BACKGROUND

Congenital anomalies are structural or functional malformations that occur at conception or during intrauterine development.^1^ Approximately 6% of births are affected each year,^2^ with congenital anomalies contributing to 2.7 million neonatal deaths globally in 2015 alone.^1^ For high‐income countries, congenital anomalies are the leading cause of perinatal death.^3^, ^4^ Beyond mortality, congenital anomalies are associated with substantial morbidity^5^, ^6^ and increased paediatric hospitalisation.^7^ However, approximately 70% of anomalies are preventable or have interventions that are lifesaving or significantly reduce disability.^2^

Prenatal diagnosis improves reproductive choices by increasing opportunities for informed decisions regarding pregnancy outcome, such as elective termination of pregnancy for fetal anomaly (TOPFA), and alternative approaches in pregnancy management and delivery.^8^, ^9^, ^10^ For many anomaly types, prenatal diagnosis is associated with reduced mortality and morbidity due to increased opportunities for intervention.

These benefits of prenatal diagnosis have led to rapid developments in imaging modalities, genetic testing and routine screening. Advances in ultrasound such as colour and power doppler have facilitated fetal echocardiography, increasing diagnostic accuracy and expanding the types of cardiovascular anomaly that can be detected prenatally.^11^, ^12^, ^13^ Three‐dimensional ultrasound has significantly improved assessment of the fetal face and skeleton,^14^, ^15^ and fetal MRI has improved the diagnostic accuracy and precision of certain nervous system anomalies of the brain.^16^, ^17^, ^18^ For functional anomalies, advances in molecular genetics have enabled chromosomal microarray analysis and non‐invasive prenatal testing (NIPT), improving the detection and diagnosis of chromosomal anomalies.^19^, ^20^

Few studies have examined changes in prenatal diagnosis over time, with most focused on a specific type of anomaly. Furthermore, only two studies have examined the age at diagnosis of congenital anomalies beyond infancy for the major types of anomaly, though neither examined changes over time.^21^, ^22^ This study describes the trends in prenatal diagnosis for the major types of congenital anomaly between 1980 and 2020 in Western Australia and examines the association between maternal and case demographics and prenatal diagnosis.

## 2 METHODS

### 2.1 Data source

This population‐based retrospective cohort study used de‐identified data from the Western Australia (WA) Register of Developmental Anomalies (WARDA). The study population included all births diagnosed with any congenital anomaly within the prenatal period and in early childhood (including TOPFA and stillbirths) from 1980 to 2020.

Since 1980, WARDA has conducted state‐wide, population‐based active surveillance of congenital anomalies for all births in WA. WARDA defines a congenital anomaly as any structural or functional anomaly present at conception or before the end of pregnancy and specifies a case ascertainment period of up to six years of age (early childhood).^23^ This period comprises diagnoses made prenatally (with confirmation at birth), after stillbirth (minimum 20 weeks' gestation), TOPFA (regardless of gestational age; confirmed after termination), or at live birth and early childhood.^23^ Mandatory notification to WARDA has been a statutory requirement since 2011, with the registry further ascertaining current and historical cases from multiple sources including the statutory WA Midwives' Notification System, the Hospital Morbidity Data Collection, and the WA Death Register. Active case ascertainment methods are used including the review of medical records from over 60 hospitals and medical facilities, diagnostic laboratories, and state screening programs.^24^

Anomalies are coded using the British Paediatric Association extension of the International Classification of Diseases Version 9 (BPA‐ICD9).^25^ To reflect severity, anomalies are coded as major or minor according to the original coding schema used by the Centers for Disease Control.^10^ A maximum of ten anomaly diagnoses per case are notified to WARDA, with the following information recorded for each diagnosis: diagnosis code (BPD‐ICD9); major/minor anomaly classification; age at diagnosis.

### 2.2 Demographic characteristics and study variables

The following information is recorded for every affected birth notified to the WARDA: birth month and year; birth postcode; birth outcome (TOPFA, stillbirth, live birth); age at death (days); sex (male, female, indeterminate, unknown); Aboriginal (yes/no); pregnancy plurality (single/multiple); mother's date of birth; multiple anomalies (single/multiple).

For analysis, individual anomaly diagnoses were categorised into groups based on site according to BPA‐ICD9 classification of diagnosis codes and in line with WARDA reporting categories.^23^ The mother's date of birth was used classify mothers into advanced maternal age (AMA), defined as over 35 years of age at the time of birth, and postcode information was classified as either metropolitan, rural or remote according to the 2001 Australian Standard Geographical Classification.^26^

### 2.3 Outcomes

For this study, the age at diagnosis was classified as: prenatal; neonatal (0–28 days); infancy (29–365 days); early childhood (1 to < 3 years); childhood (3–6 years). For age at diagnosis of all anomalies, the number of cases diagnosed with any anomaly and the age at which they were diagnosed was used to illustrate the proportion of the population affected by a congenital anomaly. Therefore, a baby was only counted once in any age at diagnosis group for estimates of all anomalies. For major anomaly types, the number of anomalies diagnosed was used to illustrate the prevalence of a major anomaly type relative to the other major types. Therefore, a baby may be counted in multiple major anomaly types and age at diagnosis groups. Anomalies diagnosed post‐mortem were assigned to the corresponding age group at which the post‐mortem was performed. For cases where no information on time of diagnosis was available (*n* = 409; 0.6%), the time of notification to WARDA was used to estimate age at diagnosis.

### 2.4 Statistical analysis

Demographic characteristics are described in counts and percentages of cases. Percentages were calculated using only cases with a recorded value, with the denominator altered accordingly for cases where data was missing or unknown. Prevalence of major anomaly types by age at diagnosis was defined as the total number of cases in live births, stillbirths, and TOPFA, divided by the number of all births in WA, expressed as the number per 10,000 births. Tabulated denominator data for all WA live births and stillbirths of at least 20 weeks' gestation during the study period were obtained from the statutory WA Midwives Notification System.^27^ Babies born after 2014 had not yet completed the full WARDA case ascertainment period (prenatal period to 6 years of age) at the time of this study, and therefore, the opportunity to diagnose late presenting anomalies was yet to occur, resulting in slightly underestimated prevalence estimates for diagnoses made in early childhood and childhood. The case ascertainment period was complete up to 2017 for diagnoses made in early childhood and in 2014 for diagnoses made in childhood. Considering this, the last complete ten‐year period of the study was 2005–2014, which was used to estimate prevalence ratios (PR) to assess the magnitude of change in prenatal diagnosis prevalence between the first (1980–1989) and last (2005–2014) ten‐year periods of the study by dividing the prevalence between the two time periods.

Joinpoint regression analysis was used to examine changes in the trend of prenatal diagnosis by identifying the years at which a change in the slope of the temporal trend occurred, as indicated by a joinpoint, which represents the best‐fitting point at which the rate of prenatal diagnosis changed.^28^ The estimated annual percentage change (APC) represents the magnitude of change in prenatal diagnosis for each year within an identified trend interval and allows the magnitude of change in trend to be compared across major anomaly types. The approach assumes that the rate of prenatal diagnosis changes at a constant percentage per year and is determined as significant if the change in slope is different from zero.^28^ Joinpoint analyses were performed for prenatal diagnosis of any congenital anomaly and for each major anomaly type for the entire study period.

Log‐binomial regression models were fit to estimate risk ratios (RR) and 95% confidence intervals (CI) for the association between demographic characteristics and prenatal diagnosis of any anomaly. A saturated multivariable log‐binomial regression analysis was then performed to estimate adjusted RR. Locally weighted scatterplot smoothing (LOWESS) was used to assess linearity between the continuous variable birth year and the logit of prenatal diagnosis, with the association demonstrated to be linear.

Prevalence ratios and 95% CI were calculated using Microsoft Excel. Joinpoint regression analyses were performed using the Joinpoint Regression Program (version 4.8.0.1^29^) and demographic characteristics, log‐binomial regression analyses and LOWESS were estimated using Stata 17.^30^

### 2.5 Missing data

For the univariable analyses, data were missing for 472 (0.7%) cases for residential region analysis, 140 (0.2%) cases for Aboriginality, 1276 (1.8%) cases for birth plurality and 551 (0.8%) cases for sex. A total of 2366 (3.3%) cases were excluded from the multivariable analysis. Complete data were available for all other analyses.

### 2.6 Ethics approval

The project was approved by the Western Australia Department of Health Human Research Ethics Committee (RGS3846) and the University of Western Australia Human Research Ethics Committee (RA/4/20/6111).

## 3 RESULTS

There were 1,132,589 births in WA from 1980 to 2020, with 91,883 congenital anomalies among 72,158 cases notified to WARDA. Of the total cases, 34,598 (57.8%) were male, 3522 (5.4%) identified as Aboriginal, 8763 (14.7%) resided rurally, and 4795 (8.0%) remotely. Most Aboriginal children with congenital anomalies were born in rural (*n* = 696; 19.9%) or remote areas (*n* = 1515; 43.4%).

Between 1980–1989 and 2011–2020, within WARDA records, stillbirths decreased from 2.0% (*n* = 251) to 0.9% (*n* = 194), live births decreased from 94.8% (*n* = 11,910) to 88.7% (*n* = 19,323), and TOPFA increased from 3.2% (*n* = 400) to 10.4% (*n* = 2271). Two or more anomalies were recorded for 29.2% (*n* = 21,044) cases and 85.7% (*n* = 61,870) of cases were diagnosed with at least one major anomaly. Over the entire study period, musculoskeletal (189.3 per 10,000), urogenital (177.3 per 10,000), and cardiovascular (114.3 per 10,000) anomalies were the most diagnosed anomaly types.

For age at diagnosis of any congenital anomaly over the study period, 16.7% (*n* = 12,064) cases were diagnosed prenatally, 36.7% (*n* = 26,457) in the neonatal period, 29.5% (*n* = 21,281) in infancy, 8.8% (*n* = 6360) in early childhood and 7.7% (*n* = 5586) in childhood. For children born in 1980, 2.3% (*n* = 25) were diagnosed prenatally, 46.8% (*n* = 500) were diagnosed in the neonatal period, 28.1% (*n* = 300) were diagnosed in infancy, 13.7% (*n* = 146) were diagnosed in early childhood and 8.5% (*n* = 91) were diagnosed in childhood. In 2014 (the latest year with all follow‐up), 22.5% (*n* = 610) were diagnosed prenatally, 26.18% (*n* = 709) were diagnosed in the neonatal period, 39.8% (*n* = 1081) were diagnosed in infancy, 7.6% (*n* = 205) were diagnosed in early childhood and 4.1% (*n* = 110) were diagnosed in childhood.

### 3.1 Rates of age at diagnosis of congenital anomaly over time

**FIGURE 1:**
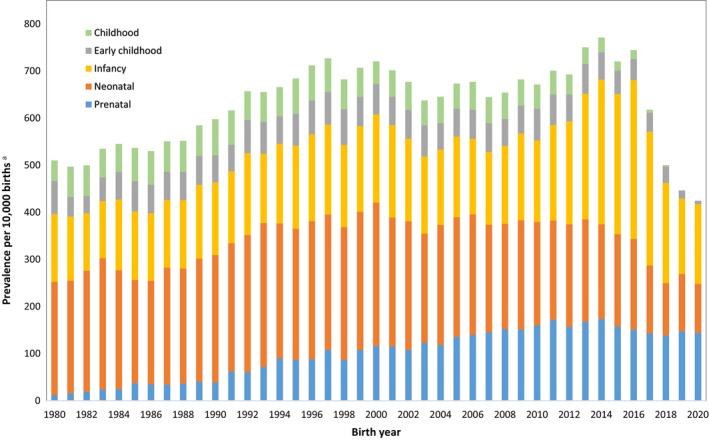
Age at diagnosis of any congenital anomaly per 10,000 births in WA between 1980 and 2020.^a^ *N* = 72,158 cases for entire study period.

Between 1980 and 2020, prenatal diagnosis of any congenital anomaly increased from 12.0 to 144.2 per 10,000 births (Figure 1). Diagnosis of any anomaly in the neonatal period decreased over the study period from 240.2 to 103.9 per 10,000 births, whilst first diagnoses made in infancy increased from 144.1 to 169.3 per 10,000 births, with notable increases from 2013 onwards. Between 1980 and 2017, diagnoses made in early childhood decreased from 70.1 to 40.5 per 10,000 births, while diagnoses made in childhood decreased from 43.7 to 31.2 per 10,000 births between 1980 and 2014.

When the first (1980–1989) and last (2005–2014) complete ten‐year periods were compared, increases in prenatal diagnosis with coinciding decreases in neonatal diagnoses and an increase in infancy diagnoses to account for late presenting anomalies were observed for all major anomaly types (Table 1). Prenatal diagnosis increased considerably for all major anomaly types, with the largest gains observed for cardiovascular (PR 10.7, 95% CI 8.0, 14.6), urogenital (PR 10.5, 95% CI 8.7, 12.6) and chromosomal (PR 7.0, 95% CI 5.9, 8.3) anomalies. Neonatal diagnosis decreased nominally for all major anomaly types except cardiovascular anomalies, and diagnoses made in infancy increased for all anomaly types with the exceptions of gastrointestinal anomalies, anomalies of the eye and of integument.

**TABLE 1:** Age at diagnosis prevalence ratio (PR) and 95% confidence interval (95% CI) for the first and last ten‐year complete case ascertainment periods of the study for all anomalies and for each major anomaly type.

	Prenatal			Neonatal			Infancy			Early Childhood			Childhood		
	Rate per 10,000 births		PR (95% CI)	Rate per 10,000 births		PR (95% CI)	Rate per 10,000 births		PR (95% CI)	Rate per 10,000 births		PR (95% CI)	Rate per 10,000 births		PR (95% CI)
	1980–1989^a^	2005–2014^b^		1980‐1989	2005‐2014		1980–1989	2005–2014		1980–1989	2005–2014		1980–1989	2005–2014	
All anomalies^c^	28.3	156.1	5.5 (5.1, 6.0)	246.1	225.0	0.9 (0.9, 0.9)	141.1	203.6	1.4 (1.4, 1.5)	56.5	61.0	1.1 (1.0, 1.2)	63.4	48.3	0.8 (0.7, 0.8)
Nervous system^d^	13.8	23.7	1.7 (1.5, 2.0)	18.4	11.0	0.6 (0.5, 0.7)	6.3	11.8	1.9 (1.5, 2.3)	4.1	5.3	1.3 (1.0, 1.7)	3.3	4.4	1.3 (1.0, 1.7)
Eye^e^	0.6	1.0	1.6 (0.8, 3.0)	6.8	4.3	0.6 (0.5, 0.8)	2.7	2.6	1.0 (0.7, 1.4)	1.1	1.4	1.3 (0.8, 2.1)	1.1	1.1	1.0 (0.6, 1.6)
Ear, face and neck^f^	1.3	7.1	5.5 (3.8, 8.0)	16.2	15.3	0.9 (0.8, 1.1)	2.7	5.5	2.1 (1.6, 2.8)	3.1	7.1	2.3 (1.8, 3.0)	2.7	4.9	1.8 (1.4, 2.5)
Cardiovascular^g^	1.9	20.7	10.7 (8.0, 14.6)	38.1	43.8	1.2 (1.1, 1.3)	28.3	42.3	1.5 (1.4, 1.6)	9.5	6.6	0.7 (0.6, 0.8)	8.9	6.5	0.7 (0.6, 0.8)
Respiratory^h^	2.2	6.5	3.0 (2.2, 4.0)	5.1	2.6	0.5 (0.4, 0.7)	0.6	1.2	2.0 (1.1, 3.6)	0.1	0.4	2.7 (0.6, 9.8)	0.2	0.3	1.5 (0.4, 4.9)
Gastrointestinal^i^	2.9	13.9	4.8 (3.7, 6.2)	35.9	24.9	0.7 (0.6, 0.8)	23.4	18.1	0.8 (0.7, 0.9)	1.5	2.2	1.4 (1.0, 2.2)	1.8	2.2	1.3 (0.9, 1.9)
Urogenital^j^	5.2	54.8	10.5 (8.7, 12.6)	70.2	70.8	1.0 (0.9, 1.1)	21.4	38.4	1.8 (1.6, 2.0)	26.2	19.3	0.7 (0.6, 0.8)	31.1	15.5	0.5 (0.4, 0.6)
Musculoskeletal^k^	6.9	35.9	5.1 (4.4, 6.1)	87.6	74.7	0.9 (0.8, 0.9)	42.0	68.4	1.6 (1.5, 1.8)	5.9	10.8	1.8 (1.5, 2.2)	3.9	5.6	1.4 (1.1, 1.8)
Integument^l^	0.3	1.2	4.7 (2.0, 11.1)	18.6	17.9	1.0 (0.9, 1.1)	7.1	7.4	1.0 (0.9, 1.3)	3.7	5.6	1.5 (1.2, 2.0)	3.3	3.6	1.1 (0.8, 1.4)
Chromosomal^m^	6.1	42.6	7.0 (5.9, 8.3)	15.7	13.5	0.9 (0.7, 1.0)	1.8	3.3	1.9 (1.3, 2.7)	1.0	3.9	3.8 (2.6, 5.9)	1.6	4.2	2.6 (1.8, 3.7)
Other^n^	6.0	21.1	3.5 (3.0, 4.3)	20.9	21.5	1.0 (0.9, 1.2)	15.6	22.5	1.4 (0.9, 1.2)	8.0	12.3	1.5 (1.3, 1.8)	10.1	9.0	0.9 (0.8, 1.1)

^a^ *N* = 12,624 cases diagnosed between 1980 and 1989. For individual anomalies, 1102 anomalies were diagnosed prenatally, 7791 neonatally, 3541 in infancy, 1500 in early childhood and 1588 in childhood between 1980 and 1989.

^b^ *N* = 8516 cases diagnosed in this period between 2005 and 2014. For individual anomalies, 7187 anomalies were diagnosed prenatally, 9446 neonatally, 6947 in infancy, 2353 in early childhood and 1799 in childhood between 2005 and 2014.

^c^ *N* = 72,158 cases for entire study period.

^d^ *N* = 5673 anomalies for entire study period.

^e^ *N* = 1286 anomalies for entire study period.

^f^ *N* = 3932 anomalies entire study period.

^g^ *N* = 12,945 anomalies entire study period.

^h^ *N* = 1186 anomalies entire study period.

^i^ *N* = 6970 anomalies entire study period.

^j^ *N* = 20,080 anomalies entire study period.

^k^ *N* = 21,436 anomalies entire study period.

^l^ *N* = 4155 anomalies entire study period.

^m^ *N* = 5704 anomalies entire study period.

^n^ *N* = 8516 anomalies entire study period.

### 3.2 Annual percent change in prenatal diagnosis for major anomaly types

**FIGURE 2:**
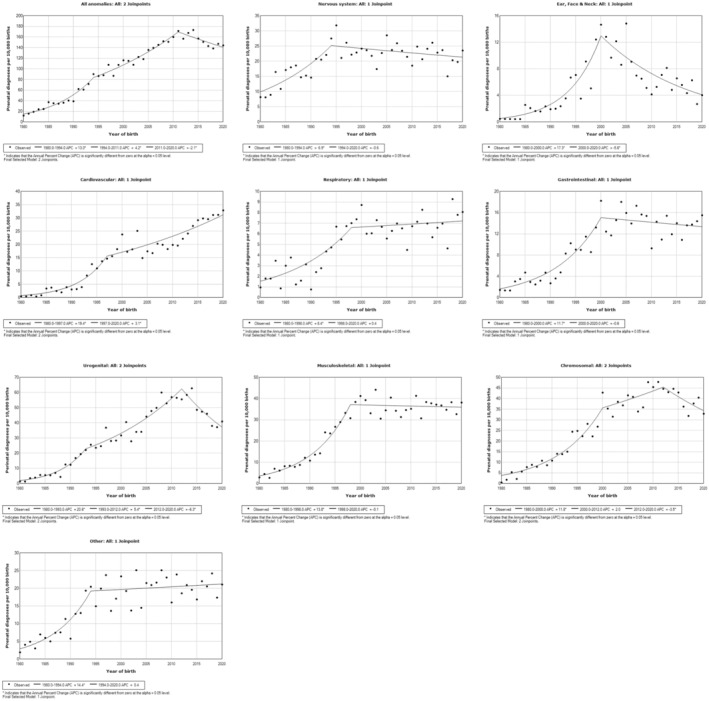
Joinpoint regression analyses for changes in trend of prenatal diagnosis prevalence (per 10,000 births) of all congenital anomalies and of each major anomaly type in WA between 1980 and 2020.

Marked increases in prenatal diagnosis were observed for all anomalies and for each major anomaly type across the first trend period, spanning 1980 to the mid‐1990s and to 2000 for some major anomaly types (Figure 2; Table S1). Substantial increases in the trend of prenatal diagnosis prevalence were observed for urogenital anomalies, cardiovascular anomalies, anomalies of the ear, face and neck and other type anomalies. In the second trend period, prenatal diagnosis prevalence continued to increase for urogenital and cardiovascular anomalies whilst prenatal diagnosis prevalence of anomalies of the ear, face and neck notably decreased. For the same trend period, prenatal diagnosis prevalence of all other major types of anomalies remained relatively unchanged. Change in trend over a third time period was identified for urogenital and chromosomal anomalies, where prenatal diagnosis prevalence was observed to decrease for both anomaly types from 2012 to 2020, as well as for prenatal diagnosis prevalence of all anomalies.

### 3.3 Association of demographic characteristics with prenatal diagnosis

In both the univariable and multivariable models, birth year, AMA, having multiple anomalies and major anomalies were associated with an increased likelihood of prenatal diagnosis. Birth plurality, remote residence and Aboriginality were associated with a decreased likelihood of prenatal diagnosis (Table 2).

**TABLE 2:** Univariable and multivariable log‐binomial regression analyses of demographic characteristics and the likelihood of prenatal diagnosis of any congenital anomaly in WA between 1980 and 2020.

	Unadjusted risk ratio (95% CI)	Adjusted risk ratio (95% CI)^a^
Birth year	1.04 (1.04, 1.04)	1.03 (1.03, 1.04)
Advanced maternal age	1.35 (1.31, 1.41)	1.14 (1.11, 1.18)
Residential region^b^		
Metropolitan	1.00 (Reference)	1.00 (Reference)
Rural	0.97 (0.92, 1.02)	1.02 (0.98, 1.08)
Remote	0.84 (0.78, 0.90)	0.89 (0.83, 0.95)
Aboriginal^c^	0.93 (0.86, 1.00)	0.90 (0.83, 0.97)
Residential region, stratified by Aboriginality		
Non‐Aboriginal		
Metropolitan	1.00 (Reference)	–
Rural	0.97 (0.92, 1.02)	–
Remote	0.92 (0.85, 0.99)	–
Aboriginal		
Metropolitan	1.00 (Reference)	–
Rural	0.87 (0.71, 1.05)	–
Remote	0.53 (0.45, 0.64)	–
Birth plurality^d^	0.96 (0.88, 1.04)	0.88 (0.83, 0.97)
Sex (female)^e^	0.98 (0.95, 1.02)	0.96 (0.93, 0.99)
Multiple anomalies	3.47 (3.36, 3.58)	2.86 (2.77, 2.96)
Major anomaly	6.18 (5.54, 6.90)	3.75 (3.36, 4.19)

^a^ *N* = 69,719 cases were included in the analyses, with 2349 cases excluded due to missing data.

^b^ *N* = 71,686. Information not recorded for 472 cases.

^c^ *N* = 72,018. Information not recorded for 140 cases.

^d^ *N* = 70,882. Information not recorded for 1276 cases.

^e^ *N* = 71,607. Information not recorded for 551 cases. Thirty‐one cases were recorded as indeterminate and 520 as not specified and were excluded from analysis.

## 4 COMMENT

### 4.1 Principal findings

Over the study period, the prenatal diagnosis prevalence increased from 28 to 156 per 10,000 births, with the most substantial gains observed for cardiovascular, urogenital, and chromosomal anomalies. This was reflected in considerable improvements in overall age at diagnosis overall and by anomaly types. Prenatal diagnosis was negatively associated with remote residence and for Aboriginal babies, consistent with previous reports of prenatal screening uptake disparities among these population groups.^31^

### 4.2 Strengths of the study

The active case ascertainment methods and statutory regulation of WARDA is among the strengths of this study. Though only made a statutory body in 2011, there has been no notable increase in the subsequent number of notifications received by WARDA,^24^ indicating the active case ascertainment methods in place prior to mandatory reporting were effective. In addition to robust data, the population‐based retrospective cohort design minimises systematic error. However, the interpretation of changes in prenatal diagnosis of major anomaly types is complicated by the fact that individual anomalies may vary substantially in severity and symptom onset, and not all anomalies are amenable to prenatal diagnosis, including patent ductus arteriosus, congenital deafness and developmental dysplasia of the hip (DDH).

### 4.3 Limitations of the data

For cases diagnosed at or after birth, there was no information available to confirm prenatal screening was performed. However, annual perinatal statistics reports from the WA Department of Health indicate that 91% to nearly 97% of women who gave birth in the state between 1994 and 2020 had received at least one ultrasound during pregnancy.^32^, ^33^ Between 2012 and 2020, the proportion of mothers in WA who had five or more reported antenatal visits ranged from approximately 93%–96%,^34^ indicating the opportunity for prenatal diagnosis was present in the majority of pregnancies.

### 4.4 Interpretation

The increases in prenatal diagnosis of congenital anomalies observed over the study period reflect vast improvements in technology and screening programs, both in Australia and worldwide. Prenatal diagnosis prevalence is a key public health indicator used by the European Surveillance of Congenital Anomalies (EUROCAT) as it allows for enhanced comparisons of prenatal diagnosis rates in contrast to the use of prenatal diagnosis proportions.^35^, ^36^

These findings are consistent with results from the United Kingdom (UK), where prenatal diagnosis increased from approximately 68 per 10,000 births between 1985 and 1988 to 138 per 10,000 births for 1997–2000.^37^ Data from the EUROCAT reported a prenatal diagnosis prevalence of approximately 90 per 10,000 births for all congenital anomalies between 2004 and 2008, compared to estimates ranging from approximately 118–152 per 10,000 births reported in this study for the same period.^35^ Between 2008 and 2012, EUROCAT estimates reported prenatal diagnosis prevalence ranging 46–120 per 10,000 births, lower than the approximate 152–157 per 10,000 births estimated in this study for the same period.^36^ However, comparisons with other reports are complicated by differences in the ascertainment period length and completeness between different registries.^10^, ^24^ The proportion of cases with prenatal diagnosis may be overestimated in some studies due to an under ascertainment of total cases.^10^, ^21^, ^22^, ^38^ The WARDA has high levels of case ascertainment and a considerably longer ascertainment period compared to most registries,^24^ including many of which comprise the EUROCAT, thereby limiting comparisons. Comparisons are also complicated by the large size and remote regions of WA, which impact access to screening services for a small portion of the population.

Over time, different screening technologies that target specific anomaly types have been introduced. This study observed changes in the trend of prenatal diagnosis associated with some of these, such as the introduction of fetal echocardiography into routine WA diagnostic practice in 1996, which led to increases in prenatal diagnosis of congenital heart disease. For chromosomal anomalies, prenatal diagnosis prevalence increased by 10 per 10,000 births following the introduction of the combined first‐trimester screen into routine WA antenatal care in 1994 and continued to increase through 2012, reflecting increasing maternal age over time and substantiating previous reports of increased prevalence of chromosomal anomalies in this population.^23^ Compared to EUROCAT estimates for 2004–2008, prenatal diagnosis prevalence for chromosomal anomalies was higher in WA at approximately 37 to 36 per 10,000 births compared to approximately 24 per 10,000 births reported from the EUROCAT.^35^ Interestingly, a decrease in prenatal diagnosis prevalence for chromosomal anomalies was observed from 2012 onwards, which coincides with the introduction of NIPT in WA for the same year.^20^ However, NIPT is not part of routine antenatal care and population disparities in uptake exist due to cost, thereby limiting the impact of NIPT on prenatal diagnosis prevalence. Instead, it is more probable that prenatal diagnosis prevalence of chromosomal anomalies was higher for the period of 2009–2015 than it was for the previous and successive five‐year periods, creating the appearance of change in trend that will likely level out over time.

Despite considerable early increases, prenatal diagnosis prevalence of musculoskeletal anomalies has remained relatively unchanged since 1998. For overall age at diagnosis, increases in diagnoses made in infancy, early childhood and childhood for the last ten‐year period of the study reflect the increased prevalence and increased late diagnosis^39^ of the most common musculoskeletal anomaly in this population, DDH. This anomaly accounted for approximately 45% of musculoskeletal diagnoses between 1980 and 1989 and increased to 75% of diagnoses in 2014.^23^ A similar pattern was observed for overall age at diagnosis for urogenital anomalies, where diagnoses in infancy increased 1.8‐fold between the first and last decades of the study. The prevalence of hypospadias, the second most common anomaly for male infants in WA, increased at an average rate of 2% each year between 1980 and 2000 in this population.^40^ These findings reflect this increased prevalence, particularly for mild cases (i.e. distal hypospadias), which comprise 84% of diagnoses in WA and are more difficult to diagnose (and, therefore, more likely to be diagnosed later) than moderate‐to‐severe cases (i.e. proximal presentation).^40^

For prenatal diagnosis of urogenital anomalies, the considerable decrease in prevalence from 2012 onwards was unexpected, as was the sustained decrease in prenatal diagnosis prevalence of anomalies of the ear, face, and neck from 2000 onwards. Both these observed decreases in trend differ from the decrease observed for chromosomal anomalies, which was likely due to a short and limited periodic decrease in anomaly prevalence and/or diagnosis. Further investigation into the prevalence and diagnosis of specific anomalies within the urogenital and ear, face, and neck anomaly types is required to explain the observed decreases in trend. For prenatal diagnosis prevalence of all anomalies, the decrease observed from 2011 onwards reflects the decrease in urogenital anomalies, which was the second most reported major anomaly type in the WA population.

For nervous system anomalies, the reduced incidence of neural tube defects (NTD), resulting from increased periconceptional folic acid intake in Australia since 1996^23^ and mandatory fortification of flour in 2009,^41^ impacts the proportion of anomalies diagnosed prenatally. NTD are more likely to be diagnosed prenatally than other nervous system anomalies, and since a large proportion of NTD are prevented through folate supplementation and fortification whilst other nervous system anomalies are not, the magnitude of improvement in prenatal diagnosis appears lessened. For overall age at diagnosis, approximately 17% of structural nervous system anomalies are not currently detectable prenatally,^42^ and most functional nervous system anomalies, excluding congenital hearing loss, are not detectable at birth.^43^

Living remotely was associated with reduced likelihood of prenatal diagnosis of any anomaly for both Aboriginal and non‐Aboriginal populations. However, the negative impact of remote residence was more pronounced in Aboriginal births, as previously reported.^31^ The association of each successive birth year with a 4% increased likelihood of prenatal diagnosis corresponds with continual screening and diagnostic advances combined with the population effects of AMA, where increased antenatal monitoring of pregnancies in older women occurs due to the known association between AMA and various structural and chromosomal anomalies.^44^, ^45^, ^46^, ^47^ Additionally, this study substantiates reports of increased prenatal detection in fetuses with major and with multiple anomalies, as the detection of one anomaly typically prompts investigation for more.^48^, ^49^

## 5 CONCLUSIONS

Improvements across all age at diagnosis groups, characterised by increases in prenatal diagnosis and infancy over time, illustrate the impact of advances in screening and diagnostics in WA and changing population demographics. The disparity in prenatal diagnosis for populations in remote regions of WA, particularly Aboriginal populations, strengthens calls for improved screening and provision of diagnostic services for these groups.

## AUTHOR CONTRIBUTIONS

EK, CM, GB and CB were involved in the initial concept and planning of the project. CM performed the analysis and drafted the manuscript under the supervision of EK and MH. All authors reviewed and contributed to the final manuscript.

## FUNDING STATEMENT

No funding was obtained for this study. EK is currently funded by a National Health and Medical Research Council Fellowship (APP1172978).

## CONFLICT OF INTEREST STATEMENT

CM, MH, GB, CB: No interests to disclose. EK: Has previously received grant funds from MundiPharma for research unrelated to this project.

## PATIENT CONSENT STATEMENT

A waiver of consent was approved by the Department of Health Western Australia Human Research Ethics Committee in accordance with the National Statement on Ethical Conduct in Human Research.

## PERMISSION TO REPRODUCE MATERIAL FROM OTHER SOURCES

Not applicable.

## Supporting information


Table S1.

## Data Availability

The data that support the findings of this study are available on request from the corresponding author. The data are not publicly available due to privacy or ethical restrictions.
