# Supplementary material for: Trends in prenatal diagnosis of congenital anomalies in Western Australia between 1980 and 2020: A population‐based study
Source: Paediatr Perinat Epidemiol. 2023 May 4;37(7):596–606. doi: 10.1111/ppe.12983 (PMC10947594; doi:10.1111/ppe.12983)
Supplement: Supplementary file 1 — Table S1. [file PPE-37-596-s001.docx]

**Supplementary Table 1**. Annual percentage change (APC) estimates from joinpoint regression analyses of changes in trend of prenatal diagnosis prevalence (per 10,000 births) of all congenital anomalies and of each major anomaly type in WA between 1980 and 2020.

|  | **Trend 1** | | **Trend 2** | | **Trend 3** | |
| --- | --- | --- | --- | --- | --- | --- |
|  | **Period** | **APC (95% CI)** | **Period** | **APC (95% CI)** | **Period** | **APC (95% CI)** |
| All anomalies | 1980-1994 | 13.1 (11.0, 15.1) | 1994-2011 | 4.3 (4.1, 5.0) | 2011-2020 | -2.1 (-3.4, -0.7) |
| Nervous system | 1980-1994 | 6.9 (4.0, 9.8) | 1994-2020 | -0.6 (-1.4, 0.1) | - | - |
| Ear, face and neck | 1980-2000 | 17.3 (12.7, 22.0) | 2000-2020 | -5.6 (-7.5, -3.7) | - | - |
| Cardiovascular | 1980-1997 | 19.4 (14.3, 24.8) | 1997-2020 | 3.1 (2.2, 4.0) | - | - |
| Respiratory | 1980-1998 | 8.4 (5.0, 11.9) | 1998-2020 | 0.4 (-1.0, 1.8) | - | - |
| Gastrointestinal | 1980-2000 | 11.7 (9.0, 14.4) | 2000-2020 | -0.6 (-1.8, 0.6) | - | - |
| Urogenital | 1980-1993 | 20.6 (15.1, 26.5) | 1993-2012 | 5.4 (4.3, 6.5) | 2012-2020 | -6.2 (-8.9, -3.6) |
| Musculoskeletal | 1980-1998 | 13.8 (11.9, 15.8) | 1998-2020 | -0.2 (-0.8, 0.5) | - | - |
| Chromosomal | 1980-2000 | 11.8 (9.5, 14.1) | 2000-2012 | 2.0 (-0.2, 4.3) | 2012-2020 | -3.5 (-6.5, -0.23) |
| Other | 1980-1994 | 14.4 (9.8, 19.2) | 1994-2020 | 0.4 (-0.5, 1.3) | - | - |
